# Continuous monitoring of endotracheal tube position with near infrared light

**DOI:** 10.1117/1.JBO.29.3.035001

**Published:** 2024-03-12

**Authors:** Tongtong Lu, Pawjai Khampang, Ahmed Beydoun, Anna Berezovsky, Rebecca Rohde, Wenzhou Hong, Bing Yu, Joseph E. Kerschner

**Affiliations:** a Marquette University, Medical College of Wisconsin, Joint Department of Biomedical Engineering, Milwaukee, Wisconsin, United States; b Medical College of Wisconsin, Department of Otolaryngology and Communication Sciences, Milwaukee, Wisconsin, United States; c Medical College of Wisconsin, Department of Microbiology and Immunology, Milwaukee, Wisconsin, United States

**Keywords:** endotracheal tube, displacement, near-infrared light, optical sensor

## Abstract

**Significance:**

Endotracheal intubation is a common approach for airway management in critically ill patients. However, the position of the endotracheal tube (ETT) may be altered during the procedure due to head movements. Accidental displacement or dislodge of the ETT may reduce the airflow, leading to moderate to severe complications, and in some cases even fatality. Therefore, timely detection of changes in ETT position in the trachea is critical to ensure immediate and intermediate interventions to maintain the ETT in the proper position. Currently, there are no widely utilized tools for real-time monitoring of ETT positions.

**Aim:**

The goal of this study is to develop a cost-effective and easy-to-use near-infrared (NIR) device, named Opt-ETT, capable of continuously monitoring the ETT position in the trachea of a patient.

**Approach:**

A side-firing fiber is attached to the side of the ETT to illuminate the trachea tissue with NIR light, and a detector board containing five phototransistors is affixed to the chest skin to measure the intensity of diffusely transmitted light. Displacement of the ETT is estimated using second-order polynomial fitting to the ratios of the phototransistor readings. Monte Carlo simulations, *ex vivo* experiment on porcine tissue, and *in vivo* experiments using a swine model have been conducted to assess the feasibility of the device.

**Results:**

The design of the Opt-ETT device has been verified by the Monte Carlo simulations and *ex vivo* experiment. The estimation of displacement from *in vivo* experiments using the Opt-ETT exhibited a high degree of agreement with that measured by a reference sensor, with a discrepancy between −1.0 to +1.5  mm within a displacement range from −15 to +15  mm.

**Conclusions:**

The Opt-ETT device provides a potentially cost-effective solution for real-time and continuous monitoring of ETT position in patient during an intubation procedure.

## 1 Introduction

An endotracheal tube (ETT) is a flexible catheter typically made of polyvinyl chloride polymer that is inserted into the trachea to protect the airway and assist in mechanical ventilation when a patient is unable to breathe on their own due to anesthesia, sedation, injury, or illness.^1^ Correct placement of the ETT is critical, with the tip of the tube ideally located 2 to 5 cm above the carina in adults, and 0.2 to 2 cm for newborn infants with a much shorter trachea.^2^^–^^4^ Misplacement of the ETT can result in moderate to severe complications, including life-threatening situations. If the tube is inserted too far distal into the airway, bronchial intubation may occur, potentially causing hypoxemia, respiratory insufficiency or other pulmonary complications when the situation is unrecognized.^5^ If the tube position is placed too proximal, or near the vocal folds, unplanned extubation may happen. This can cause emergent and less-controlled reintubation, hypoxemic injury or death if re-establishment of the airway is not accomplished quickly following extubation.^6^ Repeated intubations are associated with a series of complications, such as higher risks in laryngeal or tracheal injury and scarring, pulmonary injury, and pneumonia.^6^^,^^7^ Therefore, appropriate ETT placement is critical, and it is widely accepted that the confirmation of proper tube placement should be performed in all patients following intubation.^8^^,^^9^ For patients who are intubated outside of the operating room for a prolonged period of time, tube placement and location is generally assessed by a chest radiography (CXR).^10^ However, this confirmation of placement is a static, single-moment-in-time assessment of tube position and does not allow for immediate feedback regarding tube placement which may change over time.

Complications associated with ETT malpositioning can not only occur during the initial intubation but also can develop over a period ranging from hours to weeks after the procedure in the intensive care unit (ICU).^11^ Head movements, such as rotation, flexion, and extension, have been found to cause displacement of the ETT in a patient’s trachea.^12^ Various techniques have been proposed to secure the ETT after intubation, ranging from straps of tape or cotton string to holder devices.^13^^,^^14^ However, the displacement of ETT due to head movements remains significant even with ETT securing techniques.^12^^,^^15^ Maintaining a proper ETT position can be challenging, particularly for patients who need to be transported or transferred and for children and neonates who have shorter tracheas.^12^^,^^16^ Particularly in children, given their limited airway length, even small movements of the ETT may result in some of the aforementioned complications. An observational study of 162 infants in neonatal ICU found that adverse events occurred in 39% of intubations, including 21.4% esophageal intubations and 7% mainstream bronchial intubations.^17^ Unplanned extubation accounted for 62% of emergent intubations.^17^

After the initial placement and confirming appropriate position in the airway by radiographic means, there are imperfect systems to assess appropriate tube location. Observation of ETT markings is unreliable as the proximal end of ETT may show no change in position relative to the lip and teeth while the distal tip has already moved.^18^ Physical examinations based on auscultation and tube measurement are utilized but have been shown to be unreliable.^9^^,^^19^ Pulse oximetry is utilized and is effective in reporting oxygenation adequacy of the patient via the ETT. However, it does not provide immediate and direct feedback when an ETT is displaced as it can take several minutes for substantive declines in the oximetry reading even when no effective oxygenation is provided. This delayed response by the oximeter occurs because this device measures peripheral oxygen levels rather than what is occurring at the pulmonary level.^20^ The accuracy of pulse oximetry readings can also be affected by environmental lighting and settings of hypothermia, hypotension, poor perfusion, anemia, and nail polish.^19^ End-tidal carbon dioxide (${\mathrm{CO}}_{2}$) detection methods, including capnography and capnometry, are based on the measurement of ${\mathrm{CO}}_{2}$ concentration in exhaled gas.^19^ While these methods provide high accuracy, their effectiveness can be compromised in decreased perfusion states. In addition, these methods do not directly measure ETT position but simply provide information once the placement is comprised to such an extent as to hinder adequate ventilation. Limited availability may also prevent their use.^9^^,^^19^^,^^20^

For patients that require intubation in the ICU setting, the ideal situation would be a continuous monitoring device that would alert medical providers to a change in tube position prior to any of the difficulties noted above to allow for prevention of accidental extubation or overly distal position of the ETT. The use of ultrasound imaging for ETT placement confirmation or endoscopy has been reported; however, their limited availability, requirement for user experience, labor intensiveness, and cost hinder their use in clinical settings. Like CXR, these monitoring methods may only provide a single moment-in-time assessment of placement rather than a continuous assessment of tube position.^4^^,^^19^^,^^21^^,^^22^ Currently, SonarMed (formally named AirWave) is an FDA-approved device for real-time ETT airway monitoring for neonates. However, an earlier pilot study showed notable discrepancies in the results compared to those obtained with CXR.^23^ Novel devices based on optoacoustic imaging,^24^ magnetic field localization,^25^ radio frequency identification localization,^26^ and piezoresistive tactile sensors^27^ have been proposed, but further investigations are necessary to validate their feasibility. Therefore, there is still an unmet need for cost-effective and easy-to-use device capable of timely detection of ETT malpositioning in clinical settings.

We recently reported the proof of concept of a novel device based on near-infrared (NIR) light propagation in trachea tissue, named Opt-ETT for continuous monitoring of ETT position.^28^ Opt-ETT consists of a side-firing optical fiber attached to the ETT for trachea illumination and a detector board with two phototransistors for light detection. The NIR wavelength of 810 nm is selected for its low tissue absorption and scattering, which allows a deep penetration depth, relatively low NIR background in most operating rooms and ICUs and its compatibility with low-cost silicon-based detectors. This wavelength is also near the isosbestic point of oxy- and deoxy-hemoglobin absorption, thus is least sensitive to changes in the tissue oxygenation. Compared to our previous study, we have revised the detector design, improved the data acquisition, optimized the voltage ratio method for displacement estimation, expanded the *in vivo* validation experiments, and compared the Opt-ETT data with that obtained with a reference sensor. The modified detector board includes five phototransistors for light detection. As the tube tip moves, the distribution of NIR light intensities detected on the chest skin moves accordingly. Monte Carlo simulations and *in vivo* experiments using a swine model have been performed to validate the feasibility of the device.

## 2 Materials and Methods

### 2.1 Opt-ETT Device

**Fig. 1:**
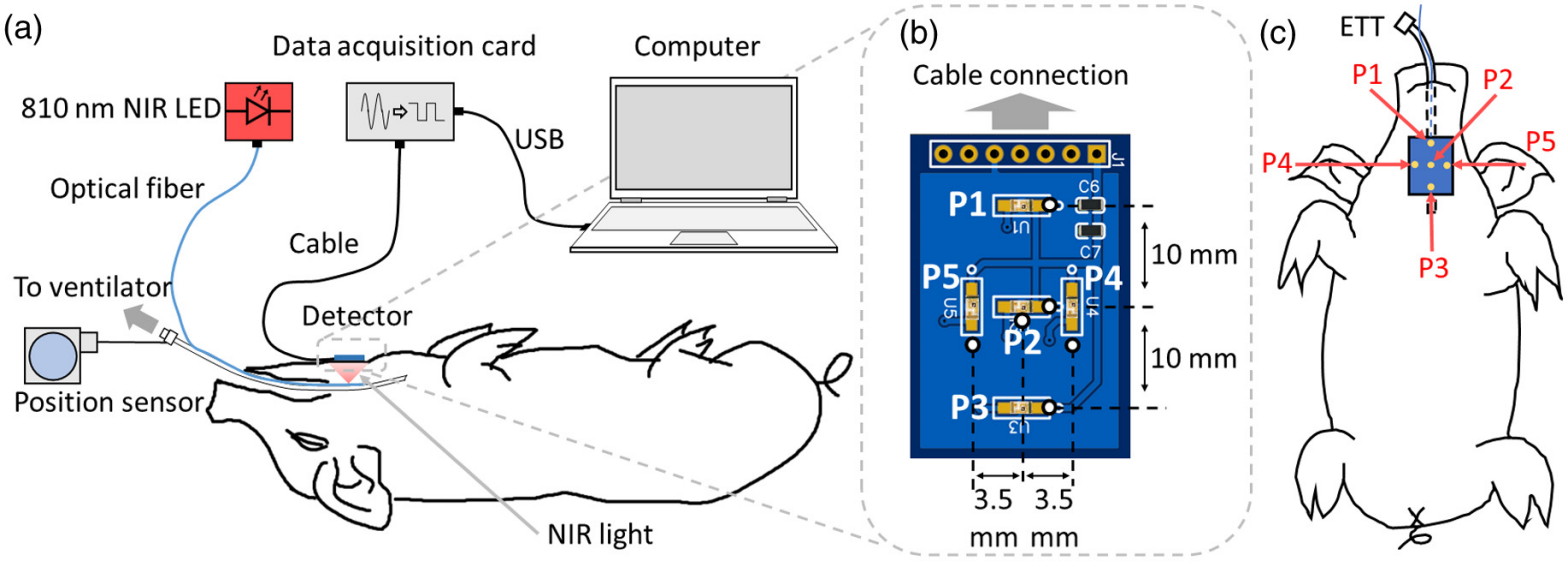
(a) Schematic of the experimental setup with an Opt-ETT device. (b) An enlarged front view of the board facing the chest skin. (c) A sketch indicating the relative positions of the sensors to the ETT. The sensors, represented by dots on the top surface of the board in the illustration, are actually located on the opposite side, facing the skin. Sensors P1–P3, placed along the ETT, are used to measure linear (longitudinal) movements of the ETT, and sensors P4 and P5 are placed perpendicular to the ETT to detect ETT rotation.

A schematic of the upgraded Opt-ETT system is illustrated in Fig. 1. The device consists of an illumination component, a detection component, and a laptop computer loaded with custom software. In the illumination component, a side-firing optical fiber with a 200  μm core diameter is attached to an uncuffed ETT, and an 810 nm NIR LED (M810F2, Thorlabs, Newton, New Jersey, United States) is used as the light source. The NIR illumination power and power density on the tissue in the current study are significantly lower than those used in the published *in vivo* studies.^29^^–^^34^ The side-firing tip of the optical fiber is approximately 1 inch from the distal end of the ETT. In the detection component, the NIR light that diffuses through the tracheal tissue and skin is captured by a detector board attached to the chest skin using a dark medical tape. The board includes five surface-mounted phototransistors P1–P5 (TEMT7000X01, Vishay Semiconductor, Malvern, Pennsylvania, United States) mounted on the front surface (skin side) of the printed circuit board, as shown in Fig. 1(b). Detectors P1, P2, and P3 are positioned with a 10 mm separation along the ETT to monitor the linear displacement of the tube, while Detectors P4 and P5 are placed on each side of P2 with a 3.5 mm separation to detect rotations of the tube. The five phototransistors are connected to a +5V power supply in a common-collector configuration. The emitter resistors (430 kΩ) determine the gain for signal amplification. A resistor-capacitor low-pass filter with an 82 nF capacitor and a 200 kΩ resistor is included in each phototransistor output, resulting in a cutoff frequency of 9.7 Hz. The output voltage signals are recorded by a data acquisition card (USB-6001, National Instruments, Austin, TX) connected to a laptop computer. The data acquisition card has a maximum sampling rate of 20 kHz; nonetheless, the sampling rate was adjusted to 250 Hz, which is sufficient for ETT displacement monitoring. A custom MATLAB (MathWorks, Natick, MA) program has been developed for real-time data acquisition and displacement calculation. During *in vivo* experiments, the connector of the ETT was attached to a resistive linear position sensor (SP1-4, TE Connectivity, Switzerland) through a thin inelastic wire. The displacement measured by the position sensor served as a reference for the Opt-ETT method.

### 2.2 Estimation of ETT Displacement

**Fig. 2:**
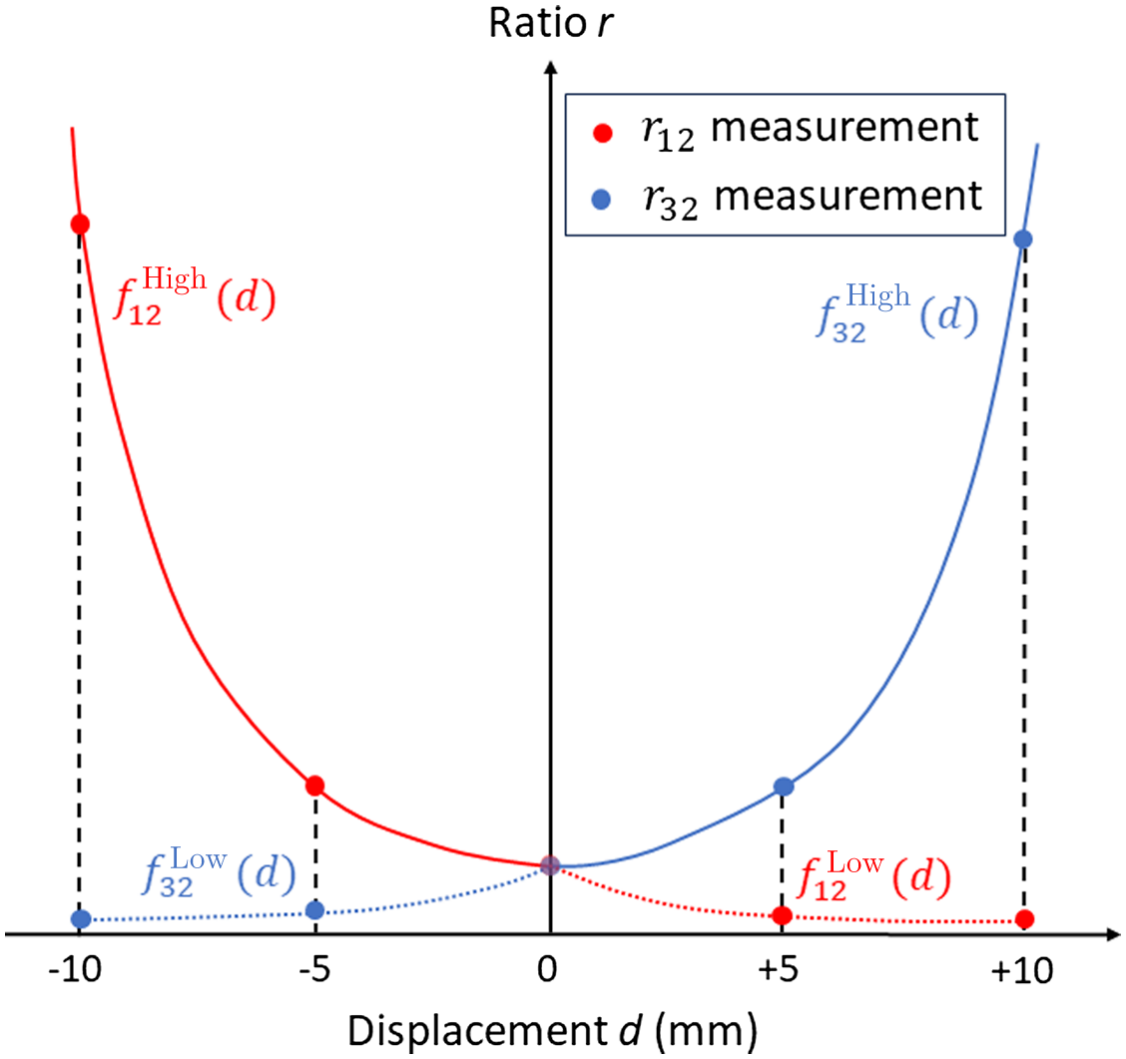
Visualization of the calibration process, which employs second-order polynomial functions, utilizing data captured at three separate displacements for each curve. The calibration curves for $r_{12}$ measurements are indicated by red lines, while the navy blue lines are the calibration curves for $r_{32}$ measurements.

The displacement of the ETT is estimated using voltages acquired from three sensors, P1, P2, and P3, denoted by $V_{P1}$, $V_{P2}$, and $V_{P3}$, respectively. Two voltage ratios, $r_{12}=V_{P1}/V_{P2}$ and $r_{32}=V_{P3}/V_{P2}$, are calculated as input parameters for an estimation model. Prior to real-time monitoring, calibration of the Opt-ETT is essential to account for the diverse thickness and properties of tracheal tissue across individuals. This calibration process involves measurements performed at five ETT locations, −10, −5, 0, +5, and +10  mm, measured by the reference position sensor. The “0” position is established by vertically aligning the tip of the side-firing fiber with the P2 sensor, as confirmed by the attainment of the maximum reading in P2. A positive displacement is induced by pushing the ETT inward, while pulling out the tube results in a negative displacement. Second-order polynomial functions are employed for calibration, which defines the relationship between voltage ratios and displacement. The visualization of the calibration procedure is illustrated in Fig. 2. In the negative displacement range, a polynomial model, $f_{12}^{\text{High}}$ (red solid line), is used to establish the relationship between $r_{12}$ and displacement d, based on calibration measurements at −10, −5, and 0 mm. Similarly, a low signal intensity model $f_{32}^{\text{Low}}$ (blue dotted line) is obtained using $r_{32}$ values measured at the three positions. In the positive displacement range, a high signal intensity model $f_{32}^{\text{High}}$ (blue solid line) and a low signal intensity model $f_{12}^{\text{Low}}$ (red dotted line) are fitted using calibration measurements $r_{32}$ and $r_{12}$ at 0, +5, and +10  mm, respectively.

For displacement estimation, the displacement direction (positive or negative) is determined by comparing $r_{12}$ (or $r_{32}$) to its values at d=0  mm $r_{12}(0)$ [or $r_{32}(0)$]. A ratio $r_{12}(d)/r_{12}(0)>1$ indicates an ETT movement towards the negative displacement (outward), whereas $r_{12}(d)/r_{12}(0)<1$ suggests an inward movement or positive direction displacement, or the opposite way if $r_{32}$ is used. As the fitted calibration curves are monotonic within their applicable ranges, displacement estimation is performed by obtaining an estimated displacement $d_{\text{High}}$ from the high signal model and $d_{\text{Low}}$ from the low signal model. The larger one of the two ratios, $r_{12}$ and $r_{32}$, is considered more reliable due to better signal-to-noise ratio (SNR) and is thus weighted more for displacement estimation. The final displacement estimation is a weighted averaging of these two estimations, formulated as $d_{\text{Final}}=w\cdot d_{\text{High}}+(1-w)\cdot d_{\text{Low}}$, where the weight w was empirically set to 0.95. The optimal weight may be further investigated in future research.

### 2.3 Monte Carlo Simulation of NIR Light Propagation in Tracheal Tissue

The operation of the Opt-ETT device relies on analyzing changes in the intensity of NIR light emitted from the skin when the tube is displaced. To assess the feasibility of the approach, Monte Carlo simulations have been performed to investigate the light distribution on the chest skin and the relationship between changes in light intensity and ETT displacement. The tissue was modeled as a five-layer structure consisting of mucosal/submucosal/glandular tissue, trachea cartridge, adipose-rich soft tissue, muscle-rich tissue, and skin, arranged from the lumen of trachea to the anterior direction, respectively. Each layer is assumed to be uniform. The optical properties and thicknesses of each layer were determined using a frequency-domain NIR spectroscopy instrument and other relevant studies.^31^^,^^35^^–^^37^ A summary of tissue properties used for the simulation is provided in Table 1. The ValoMC, an open-source MATLAB toolbox for light transport simulation developed by Aleksi Leino et al., was employed to conduct the simulations.^38^ A two-dimensional (2D) simulation was implemented to study the light intensity distribution along the direction of the sensors P1–P3. A total of 30 million photons were launched into the tissue as a point source following a cosine intensity distribution. Based on the results from the 2D simulation, voltage ratios at various tube displacements (from the 0 position with the fiber tip right below sensor P2) were calculated. The efficacy of the calibration using a second-order polynomial fitting with the five data points was evaluated using the simulated data. Furthermore, three-dimensional (3D) simulations were also performed to investigate the effect of tube rotation. The light source, i.e., the tip of side-firing fiber, was modeled with three launching angles, including 0 deg, −40  deg, and +40  deg. The light beam contained 50 million photons. The calculated voltage ratios were compared among the three launching angles.

**Table 1:** Summary of optical properties and thickness of tissue layers used for simulation.

Tissue type	Thickness (mm)	Absorption coefficient $\mu_{a}$ (${\mathrm{cm}}^{-1}$)	Scattering coefficient $\mu_{s}$ (${\mathrm{cm}}^{-1}$)	Anisotropy g	Refractive index n
Skin	0.5	1.80	408	0.95	1.36
Muscle-rich soft tissue	6.5	0.35	83	0.90	1.40
Adipose-rich soft tissue	6.5	0.22	119	0.79	1.40
Trachea cartridge	1.0	1.00	100	0.92	1.45
Mucosal/submucosal/ glandular tissue	1.5	0.70	173	0.95	1.40

### 2.4 *Ex Vivo* Experiment

**Fig. 3:**
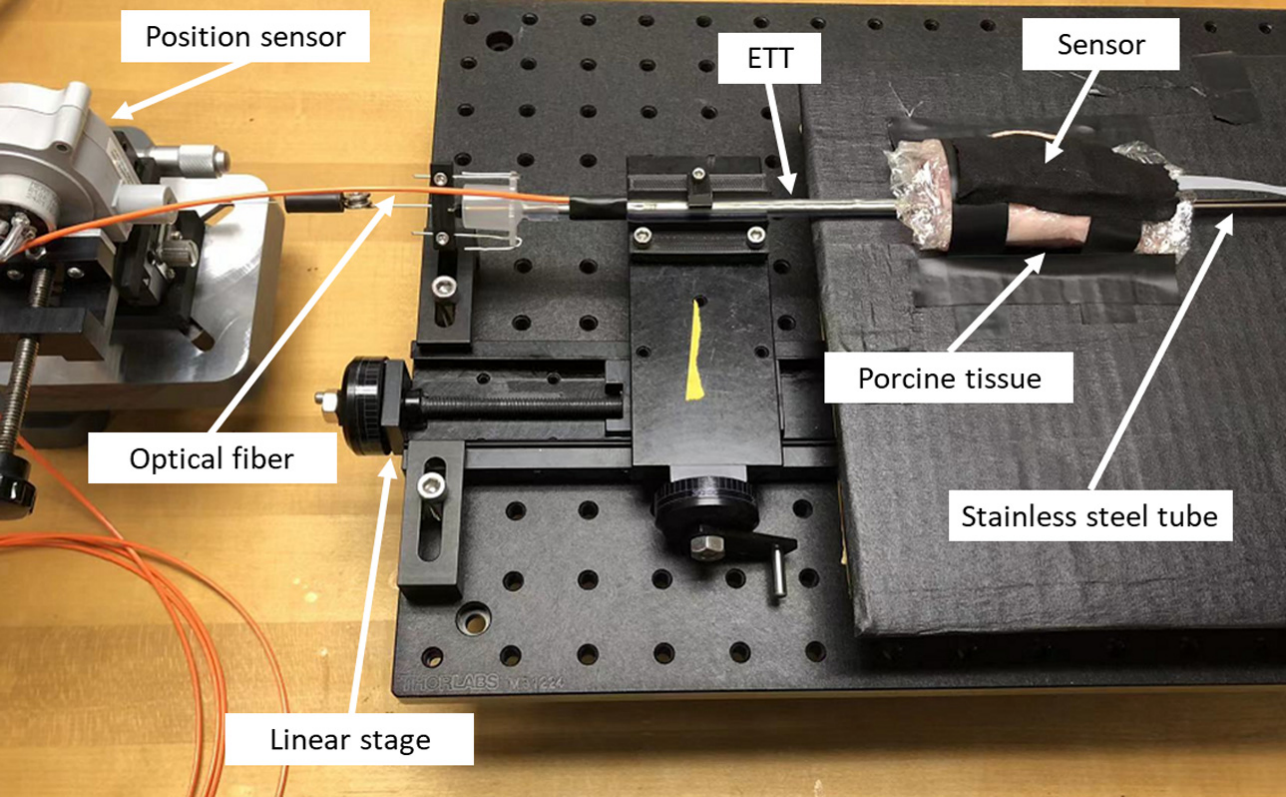
Photograph of the *ex vivo* experimental setup.

*Ex vivo* experiment was conducted on porcine tissue to assess the accuracy of the Opt-ETT device within a meticulously controlled laboratory environment. The tissue, consisting of both adipose and muscle, was approximately 16 mm thick. To prevent dehydration, the tissue surfaces were covered in clear plastic food wrap, which permits transmission of NIR light. The experimental setup for *ex vivo* experiment is shown in Fig. 3. A stainless-steel tube was inserted into the ETT, which was then attached to a linear stage, which restricts the movement of the ETT exclusively to the longitudinal axis. In a configuration similar to that illustrated in Fig. 1(a), a reference position sensor was attached to the proximal end of the ETT. The ETT was placed beneath the tissue, with its side-firing tip facing up, while the detector board, with sensors downward-facing, was attached atop the tissue. This setup was designed to simulate the scenario where an ETT is inserted into the trachea. It should be noted that, to prevent cable twisting during the experiment, the detector board was oriented such that sensors P1 and P3 were reversed along the longitudinal axis in contrast to that in Fig. 1(a). This necessitated minor modifications, specifically the interchange of data between these two sensors, of the displacement calibration and estimation procedures. The ETT was repeatedly maneuvered within a range of ±15  mm for a total of six cycles using a manual linear stage. The estimated displacement from the Opt-ETT was then compared with that of the reference sensor through scatter plot.

### 2.5 *In Vivo* Experiments

**Fig. 4:**
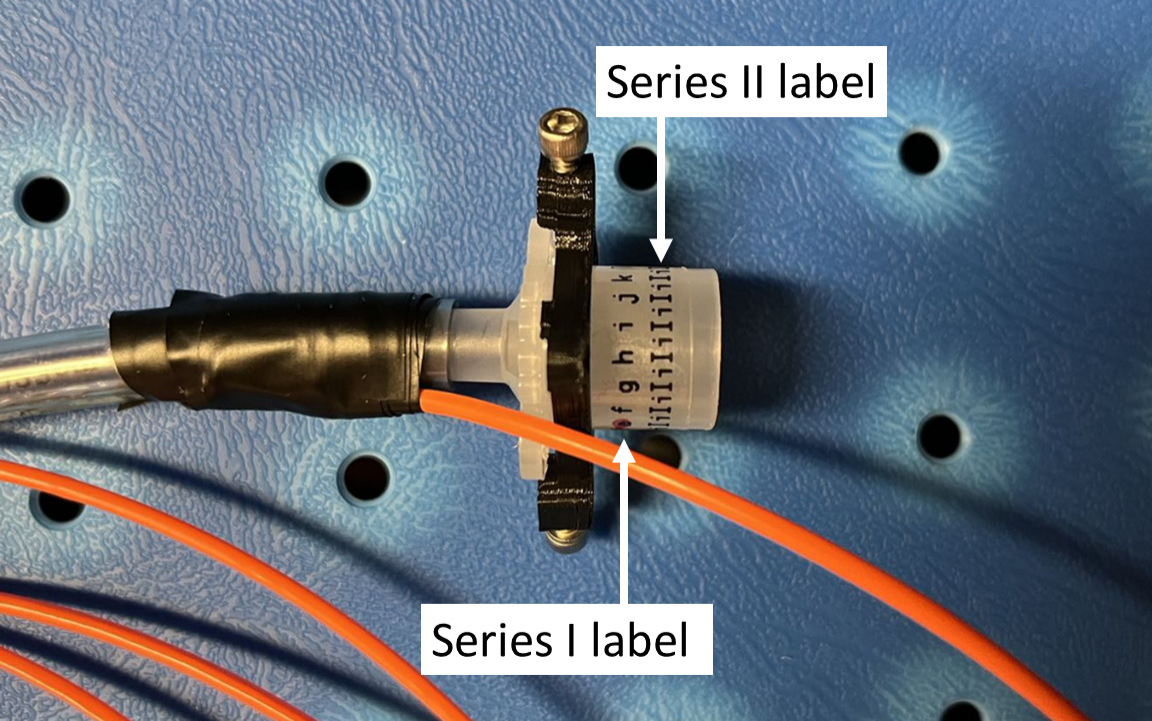
Photograph of the water-resistant alphabetical labels for rotation control during the *in vivo* experiments.

The feasibility and performance of the device were evaluated through *in vivo* experiments on eight piglets. The study was approved by the Institutional Animal Care and Use Committee of the Medical College of Wisconsin (MCW). Preparation procedures, including anesthetic injection and ETT intubation, and vital signs monitoring were conducted by MCW Biomedical Resource Center veterinary staff (Vet). Animals were measured on three different days at a mean weight of 7.8±1.3, 10.2±1.0, and 11.0±1.4  kg, respectively. The second experiment (day 14) was performed 2 weeks after the first experiment (day 1), and the third experiment (day 21) was 1 week after the second experiment, resulting in a total of 24 experiments. Prior to each experiment, the ETT tip was placed near the middle of the trachea of the animal determined by the Vet, which was set as the 0 position. The Opt-ETT detector board was taped on the chest with sensors P1–P3 aligned along the tube and P2 right above the tip of the side-firing fiber and at 0 fiber rotation (confirmed by maximum signal for P2). At the beginning of each experiment, calibration was performed using data captured at five tube locations (−10, −5, 0, +5, and +10  mm). After that, the ETT was manually displaced within a ±15  mm displacement range from the 0 position, with a step size of around 1 mm measured by the reference position sensor, and the voltages of the sensors P1–P3 were recorded by the laptop. The illumination fiber was kept facing the detector board (0 rotation position) during these procedures. Each back-and-forth movement of ETT in the full range from −15 to 15 mm is called one cycle. A summary of cycles for the experiments is presented in Table 2, with an asterisk (*) indicating that tube displacement was obtained by directly manually pulling/pushing the tube, while in the remaining experiments displacement was achieved manually but through a linear stage. To evaluate the effects of ETT rotations on the ratios $r_{12}$ and $r_{32}$, measurements were repeated with the tube rotated to two specific angles, ±40  deg. Rotation of the ETT was facilitated by affixing two sets of water-resistant alphabetical labels onto its connector, illustrated in Fig. 4. The first series of labels (“a,” “b,” “c,” etc.) corresponds to 20-deg increments, while the second series (“I” and “i”) indicates 10-deg intervals. Aligning the ETT with two specific labels from the first series enabled rotational adjustments of approximately ±40  deg.

**Table 2:** Summary of movement cycles for linear displacement. The symbol * indicates that the tube was manually pulled/pushed without using the linear stage.

Swine No.	Day 1 (cycle)	Day 14 (cycle)	Day 21 (cycle)
1	6^*^	5^*^	2^*^
2	2.5^*^	5^*^	6^*^
3	5^*^	5	5
4	5.5^*^	6	5
5	5	5	5
6	6.5	5	5
7	5	5	5
8	5	5	5

**Fig. 5:**
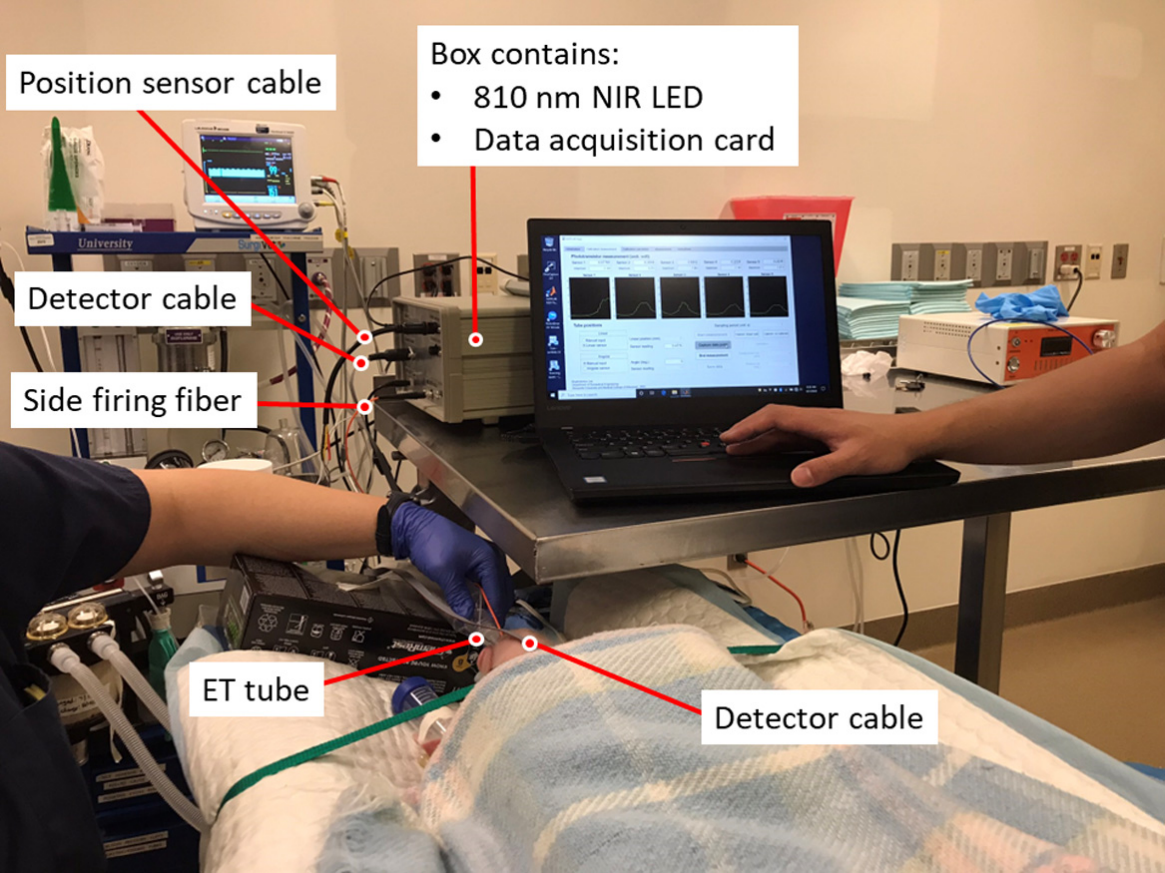
Photograph of the experimental setup for manual pull/push during the first eight experiments.

During the first eight experiments, the tube was directly pushed or pulled by the Vet or an otolaryngology resident. A picture of the experimental setup is shown in Fig. 5. Although care was taken during tube movement, tube rotations, tilting, and hand tremors might have occurred due to arm tiredness. Therefore, during the remaining 16 experiments, the connector of ETT was fixed to a linear stage for easy and stable adjustment of the tube position. In all experiments, displacements were estimated from the Opt-ETT readings using the calibrated polynomial model and compared to readings of the reference sensor using linear regression, and the degree of agreement between the two methods was investigated using the Bland-Altman method.^39^

## 3 Results

### 3.1 Monte Carlo Simulation

**Fig. 6:**
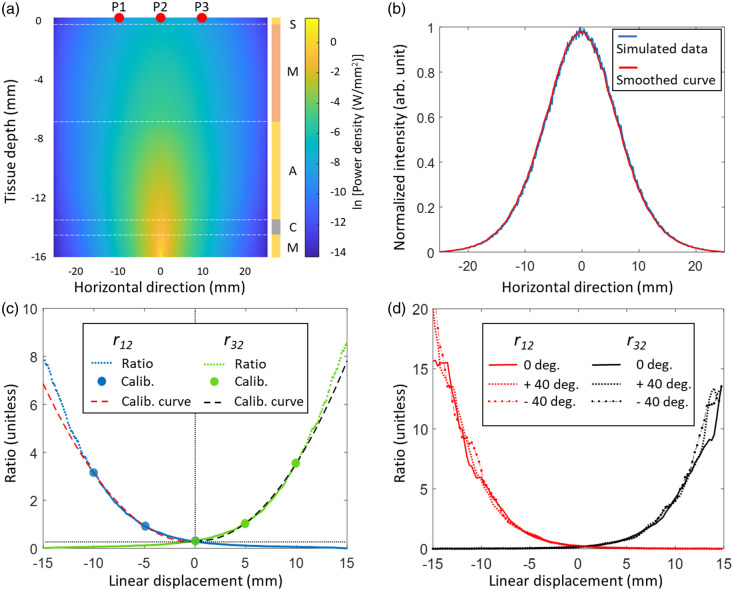
The results of Monte Carlo simulations. (a) 2D distribution of power densities in a five-layer tissue model, with “M,” “C,” “A,” “M,” and “S” representing mucosal/submucosal/glandular tissue, trachea cartridge, adipose-rich soft tissue, muscle-rich soft tissue, and skin, respectively. The red dots indicate the positions of sensors P1–P3 at the zero position. (b) Light intensity distribution on the skin in the horizontal direction (along P1–P3) from the 2D simulation. (c) Simulated voltage ratios $r_{12}$ and $r_{32}$. Sensor P2 is placed at (0, 0) position as the starting point. The dashed lines indicate the calibration function fitted by the calibration measurements denoted by the color dots. (d) Voltage ratios obtained from 3D simulations with the light source being rotated by 40 deg in both directions.

Simulation results for the propagation of NIR light in tracheal tissue are presented in Fig. 6. The 2D distribution of photon power density when the fiber tip is placed at the 0-horizontal position on the inner surface of the trachea, or (0,−16), is shown in Fig. 6(a). The white dashed lines indicate the boundaries of each tissue layer, and the tissue types are indicated in the color bar on the right. The tissue model is 16 mm thick, and the output power of the light source is normalized to 1 W in simulations for simplicity. The distribution of power density is displayed in colors with the scale based on natural logarithm. The light intensity distribution on the skin is plotted along the horizontal direction (P1–P3) in Fig. 6(b), with the smoothed result obtained using averaging filtering with 6 data points. The intensity decreases to half from the peak value at approximately ±7.5  mm. The simulated voltage ratios in Fig. 6(c) indicate that $r_{12}$ (dotted blue line) is larger than $r_{32}$ (dotted green line) in the negative displacement (ETT pulled out) range, and the trend is reversed in the positive displacement (ETT pushed in) range. The second-order polynomial model fitted ratio $r_{12}=f_{12}(d)$ (dashed red line) provides a good approximation to the Monte Carlo simulated ratio $r_{12}$ in the displacement range from −12.5 to 0 mm. Similarly, the model fitted $r_{32}=f_{32}(d)$, shown as a dashed black line, matches well with the simulated ratio $r_{32}$ in the displacement range from 0 mm to +12.5  mm, which confirms the choice of the larger voltage ratio for displacement estimation. This observation has demonstrated the feasibility of applying second-order polynomial function and five-point measurement for calibration. The voltage ratios obtained from the 3D simulations are shown in Fig. 6(d). No apparent difference was observed among the ratios between the three different rotation angles. Therefore, it is reasonable to assume that the fitted models are relatively insensitive to rotations of the ETT within ±40  deg. If the ratios P4/P2 and P5/P2 go beyond a preset value, a warning signal can be generated so that a correction of excessive rotation can be performed timely for better accuracy in displacement estimation.

### 3.2 *Ex Vivo* Experiment

**Fig. 7:**
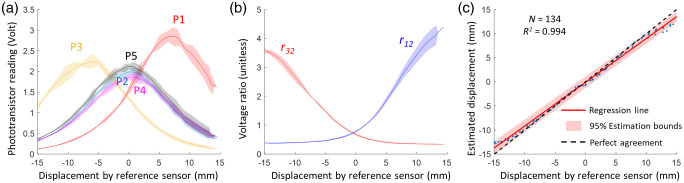
Results of the *ex vivo* experiment. (a) Voltage readings from sensors P1–P5 against displacement by the reference sensor. (b) Calculated voltage ratios $r_{12}$ and $r_{32}$ against displacement. (c) Scatter plot depicting the correlation between the estimated displacements from Opt-ETT and readings from the reference sensor. The shaded areas represent 1.96*SD or a 95% percentile with a normal distribution.

The results of the *ex vivo* experiment are shown in Fig. 7. Voltage readings from sensors P1–P3, plotted against the displacement recorded by the reference position sensor [Fig. 7(a)], exhibit a similar shape to the simulated curves depicted in Fig. 6(b). The observable difference in peak voltages of P1–P3 may be attributed to variations in the thickness and composition of the adipose and muscle tissues within the porcine sample as well as sensitivity of the three sensors. Correspondingly, the voltage ratios, as plotted in Fig. 7(b), show a trend akin to the simulations illustrated in Fig. 6(c), which suggests the feasibility of a second-order polynomial fitting for effective calibration. A total of 134 measurements were obtained. The scatter plot in Fig. 7(c) shows a high correlation ($R^{2}=0.994$) between the displacements estimated via the Opt-ETT and readings measured by the reference position sensor. This linear regression trajectory closely aligns with the line of perfect agreement. The 95% limits of agreement span from −2.5  mm to 2.8 mm across the full displacement range from −15  mm to +15  mm. The measurement data predominantly resides within these bounds, with only a few outliers occurring at displacement ranges exceeding ±11  mm, which are outside of the calibration range of ±10  mm. These *ex vivo* experiment results demonstrate a substantial level of agreement between the measurements obtained from the Opt-ETT and reference position sensor, thereby confirming the efficacy of the Opt-ETT device in a controlled experimental situation.

### 3.3 *In Vivo* Experiments

**Fig. 8:**
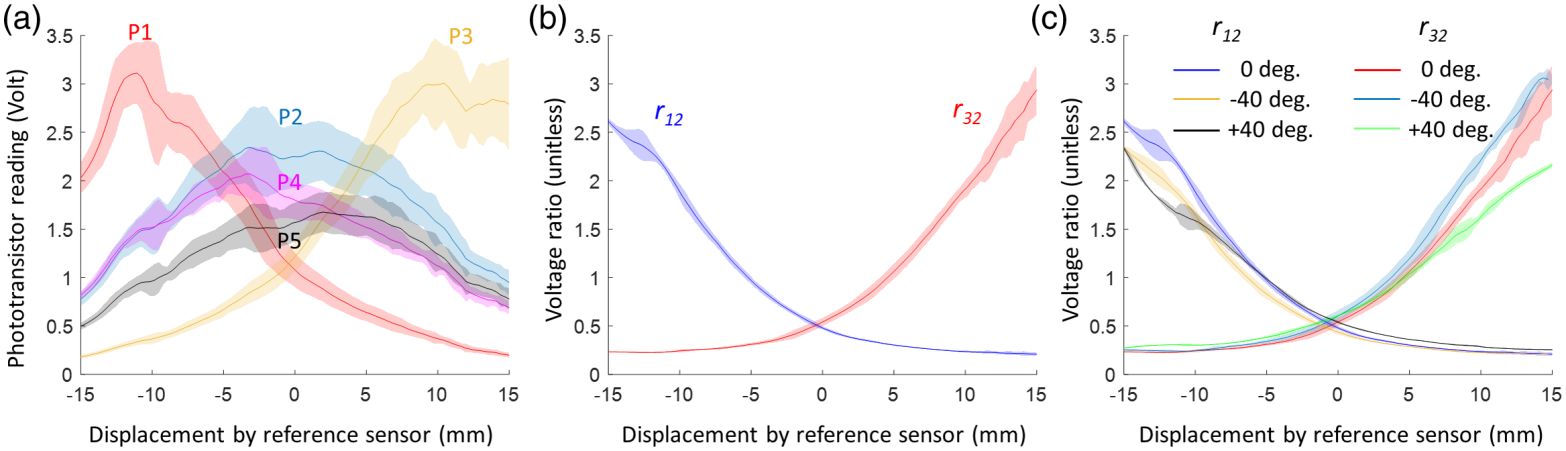
Example measurements from a single swine experiment. (a) Readings from sensors P1–P5. (b) Calculated voltage ratios $r_{12}$ and $r_{32}$. (c) Voltage ratios obtained at ETT rotations of 0 deg and ±40  deg. The shaded areas represent 1.96*SD or a 95% percentile with a normal distribution.

The results from one of the swine experiments (the day 21 experiment for Pig # 4) are presented in Fig. 8. The voltages from sensors P1–P5 are plotted against the ETT displacement measured by the reference sensor in Fig. 8(a). The tube was moved back and forth for five cycles and linear interpolation was applied to the consecutive measurements at 1.0 mm interval in displacement. The solid lines in the figure represent the mean of all measurement points, while the shaded areas indicate 1.96 times the standard deviation (i.e., 1.96*SD). The maximum voltage varies among the sensors, with P1 and P3 exhibiting peak voltages reaching 3 V, while P2 has a value below 2.5 V. This variation in maximum voltage may be attributed to the nonuniformity of the tissue, sensor responses, and the varying contact pressure between the fiber tip and the tracheal wall. The peak values for P4 and P5 are lower because the two detectors were placed 3.5 mm away from path of the fiber tip. The shape of the curves appears wider (or broader) compared to the simulated data [Fig. 6(b)], and the peak voltages may not occur exactly at 0 mm for sensors P2, P4, and P5, or at ±10  mm for P1 and P3, again likely due to tissue heterogeneity. The calculated voltages ratios $r_{12}$ and $r_{32}$ are plotted in Fig. 8(b), which follow the similar trend as the simulated data [Fig. 6(c)]. The shaded area is narrow in the displacement range of −10 to +10  mm, indicating that ratios $r_{12}$ and $r_{32}$ can be used to obtain more accurate estimate of the tube position within this displacement range than using the individual readings. The voltage ratios obtained at ETT rotation angles of ±40  deg and 0 deg are plotted in Fig. 8(c). The differences in voltage ratios among the three rotation angles are small between −10 and +10  mm, suggesting that the model calibrated at 0 deg may be directly used for measuring ETT displacement even at small to moderate angles of rotation.

### 3.4 Comparison with the Reference Sensor

**Fig. 9:**
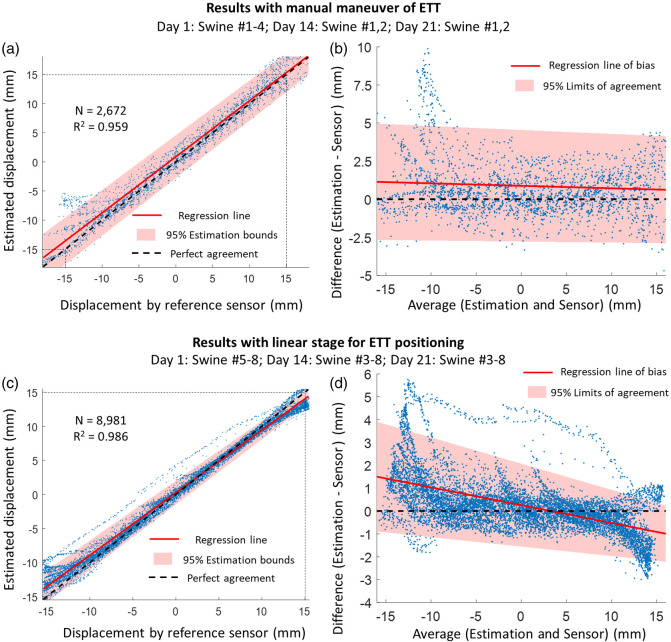
Estimated Opt-ETT displacements versus readings from the reference position sensor for all pigs. For experimental results obtained with manual maneuver of the tube, the scatter plot and Bland–Altman analysis are shown in panels (a) and (b). For experimental results obtained with a linear stage for tube positioning, the scatter plot and Bland–Altman analysis are shown in panels (c) and (d). The shaded area represents the 95% confidence interval bounds of estimation.

The comparison of displacements estimated by the Opt-ETT device with readings from the reference position sensor is shown in Fig. 9. Specifically, results obtained from experiments with manual push/pull of the ETT are shown in Figs. 9(a) and 9(b), while results obtained from experiments through the linear stage for positioning are shown in Figs. 9(c) and 9(d). In the first eight experiments with manual push/pull, the estimated displacements are plotted against those obtained with the reference sensor in Fig. 9(a). The linear regression line closely aligns with the perfect agreement line, with a coefficient of determination of 0.959, indicating a high correlation between the two methods. In Fig. 9(b), the result from the Bland–Altman analysis is presented, with the mean and difference of displacements measured by the two methods plotted on the x-axis and y-axis, respectively. The distribution of measurement points is non-uniform and shows a decreasing trend, suggesting that the measurement variances are not constant. Therefore, a linear regression is applied to estimate the bias, as shown by the red solid line. A positive bias of less than 1.25 mm is observed in the displacement range of −15 to +15  mm, indicating that the Opt-ETT approach tends to overestimate displacements slightly to the positive direction (ETT moving in).

In the sixteen experiments using the linear stage, data from one experiment was discarded due to obvious errors in the calibration measurements. The scatter plot in Fig. 9(c) shows a similar regression trend as in Fig. 9(a), but with a higher coefficient of determination of 0.986 and a tighter bound for a 95% confidence interval. The Bland–Altman analysis in Fig. 9(d) shows a decreasing bias with the mean displacement, ∼1.5  mm at −15  mm and −1.0  mm at +15  mm. The estimated bias is close to zero near 3 mm mean displacement. The 95% limit of agreement is narrower compared to those in Fig. 9(b) and exhibits a shrinking trend, resulting in less variance and uncertainty in the positive displacement range.

Overall, a high level of association was observed between the displacement obtained by the reference position sensor and the Opt-ETT device, regardless of the ETT maneuvering technique. Nevertheless, less variance and uncertainty were achieved using a linear stage for ETT positioning compared to direct manipulation by hand, which is reasonable as a linear stage is more stable and less prone to unintentional movements. It is important to note that the Bland-Altman analysis has some strict assumptions that may not always be fully fulfilled in practice, and therefore, the results sometimes can be misleading and should be interpreted with caution.^40^ Patrick Taffé has proposed a method to address this issue, but it requires repeated measurements from at least one method, which were not available in this study.^40^

## 4 Discussion

ETT intubation is crucial for providing mechanical ventilation for critically ill patients. Nonetheless, during surgeries under anesthesia or substantial sedation, or in the ICU, unnoticed ETT movement may result in moderate to severe complications, or even life-threatening situations. Therefore, proper placement of the ETT in the trachea should be confirmed immediately after insertion, and continuous monitoring of its position would provide valuable information in preventing complications arising from unnoticed ETT movement. Currently, there is no effective tool widely adopted for this purpose, and delays in detecting incorrect positioning or dislodgement of the ETT can lead to severe morbidity for patients and even death. To address this clinical issue, we have developed an Opt-ETT device that provides a prompt, safe, and convenient method of real-time assessment of ETT position. The detection board is equipped with five sensors, compared to the two sensors in our previous design, which expands the capability for higher detection sensitivity for linear displacement, larger displacement range, and better tolerance to angular rotation of the ETT. Additionally, the new detector board is significantly smaller, thinner, and lighter, making it easier to be attached to the chest skin, and thus alleviating potential loose contact between the skin and sensors. Data acquisition has also been upgraded from an Arduino board to the NI USB-6001 device, enhancing the resolution to 14 bits and providing higher sampling rates for improved speed and accuracy. Methodologically, we have shifted to using polynomial fittings based on readings from three sensors over a 20 mm range, improving noise robustness and measurement range. This study also expanded the *in vivo* experiments from one to eight piglets, allowing for a thorough evaluation of the Opt-ETT device. Additionally, we have presented a more detailed comparison of estimated displacements against reference sensor readings, utilizing scatter plots and Bland–Altman analysis, along with linear regression to assess accuracy, which is a significant enhancement over our previous study’s methodology. Moreover, this new device shares many advantages of our previous device, including no need for additional clinical personnel, simple and convenient to use, and cost-effective to manufacture. Furthermore, the detection board uses all surface mount components, which can be mass produced at an even lower cost.

Compared to the FDA-approved AirWave system, the Opt-ETT device exhibits a higher degree of agreement with the reference method. Specifically in the small but more clinically useful displacement range from −10 to +10  mm, the discrepancy between the Opt-ETT device and the reference sensor is within ±2.5  mm for most measurements. In contrast, a clinical study involving AirWave in human patients reported a discrepancy larger than 10 mm between the system’s estimation and the ETT distance at the lips for the same displacement range, highlighting the exceptional accuracy of the Opt-ETT device.^23^

The discrepancy between our device and the reference method slightly increases when the displacement exceeds ±10  mm, with an underestimation tendency at larger displacement. This may be attributed to the estimation method, which uses second-order polynomial fitting as reflected in the simulated ratios curve in Fig. 6(c). The fitted curves are consistently lower than the voltage ratios as the displacement exceeds ±10  mm, leading to an underestimation by the model. A more complex fitting model may address this issue. However, there is a trade-off between estimation precision and the burden of calibration, as a more complicated model may require additional calibration measurements due to more parameters to fit for, which may lengthen the procedure. In addition to this systematic error, low SNR in the large displacement range due to reduced light also contributes to more estimation errors.

The simulation and experimental results shown in Figs. 6(b) and 8(a) reveal noticeable differences in the voltage curves. There are several factors that may have contributed to these differences. First, the simulated tissue structure may not accurately reflect the actual tracheal and skin tissues due to variations in their thickness and optical properties of each layer, while the simulated tissue was assumed to be uniform within each layer. Second, accurately measuring optical properties of live swine tissues can be challenging. Studies have shown that measurements of optical parameters from the same tissue type can vary significantly.^37^ However, errors in the optical properties can only affect the accuracy of the Monte Carlo simulations that are used for verification of the second-order polynomial model and will unlikely change the general trend of the ratios. Finally, changes in contact between the fiber tip and the tracheal wall, or between the sensors and the skin, can lead to fluctuations in the detector readings. These changes may not be discernible in the voltage ratios, as the readings of all sensors may increase or decrease simultaneously, as observed in Fig. 8(b). Despite the existence of disparities between simulations and experiments, the general trend in the sensor voltages and the feasibility of estimating displacement using voltages ratios have been substantiated by the Monte Carlo simulations.

There are several limitations that need to be acknowledged in this study. First, the absence of a gold standard for ETT displacement and the reliance on the agreement with the position sensor used as reference may have introduced some uncertainty. The displacement obtained by the reference sensor, similar to the measure of ETT distance at the lips, may have discrepancies compared to displacement obtained from CXR.^23^ In addition, the precision and variance of the position sensor used in this *in vivo* study have not been independently confirmed, and the Bland–Altman method, which was employed for comparison, is not intended to compare a simple method with a highly precise method.^41^ Second, the displacement range in this study was limited to ±15, but this range is sufficient for pediatric patients who have a much shorter trachea and a displacement beyond 5 mm should be corrected. Third, although the sensors P4 and P5 were designed to detect ETT rotations at large angles, the alert threshold determined by these two sensors was not fully investigated, which may affect the accuracy of the device when large rotations occur. In addition, the compatibility of the device with cuffed ETTs was not studied. Cuffed ETTs can change the contact between the fiber tip and the tracheal wall, resulting in different patterns of NIR light at the skin surface. Therefore, additional simulations and experiments are necessary to evaluate the feasibility of the device with cuffed ETTs. Finally, during the placement of the ETT through the trachea, the side-firing tip of the ETT may become coated or contaminated with mucus or blood. However, such minimal residual mucus or blood is not a significant concern, as NIR light can easily penetrate mucus, and the effect of blood contamination is negligible compared to the light attenuation caused by the tissue between the trachea and skin.

## 5 Conclusions

The NIR-based Opt-ETT aims to address the unmet clinical need for a safe, cost-effective, and convenient method for continuous assessment of ETT positions during a clinical procedure where intubation is needed. This device consists of a side-firing optical fiber attached to an ETT for NIR illumination, a small detector board that can be easily taped on to the chest, and a data acquisition card connected to a laptop for real-time data collection and displacement estimation. Monte Carlo simulations of light propagation in tracheal tissues and *in vivo* experiments using swine models have demonstrated the Opt-ETT device for real-time assessment of ETT displacement with reasonable accuracy. The Opt-ETT device has the potential to prevent complications, improve patient safety, and enhance clinical outcomes during intubation.

## Data Availability

The data that support the findings of this article are not publicly available at this time but may be obtained from the authors upon reasonable request.
